# Gendered Social Norms Change in Water Governance Structures Through Community Facilitation: Evaluation of the UPWARD Intervention in Tanzania

**DOI:** 10.3389/fsoc.2021.672989

**Published:** 2021-07-05

**Authors:** Jacob Eaton, Aditi Krishna, Christina Sudi, Janeth George, Christopher Magomba, Anne Eckman, Frances Houck, Hannah Taukobong

**Affiliations:** ^1^ Iris Group, Chapel Hill, NC, United States; ^2^ Tetra Tech, Morogoro, Tanzania; ^3^ School of Agricultural Economics and Business Studies (SAEBS), Sokoine University of Agriculture, Morogoro, Tanzania

**Keywords:** gender, social norms, water governance, community engagement, organized diffusion

## Abstract

**Background:** In rural Tanzania, women and girls disproportionately bear the burden of water scarcity. Gendered social norms on the acceptability of women’s participation in the public sphere limit their decision-making power within local water governance structures. The UPWARD (Uplifting Women’s Participation in Water-Related Decision-Making) intervention sought to understand how a community-based gendered social norms approach using organized diffusion can lead to changes in the gendered social norms impacting women's participation in water-related governance structures.

**Methods:** As part of WARIDI, a 5-years integrated water resource management (IWRM) program, a gendered-social norms change (GSNC) activity (UPWARD: Uplifting Women’s Participation in Water-Related Decision-Making) was implemented in two villages in Iringa and Kilombero districts. Encouraging organized diffusion, UPWARD promoted gender-equitable norms among a critical mass of community members. WARIDI identified and trained a Community Facilitation Team (CFT) of three women and men to lead a series of education and empowerment sessions in two communities. The intervention reached >300 individuals directly (∼10% of total village population). Changes in social norms were assessed through social norms analysis plots (SNAP) delivered in focus group discussions (FGDs) of 8–12 participants.

**Results:** At baseline, most participants reported that women’s involvement in water-related decision-making was restricted to household decisions. Men viewed themselves as primary decision-makers in water governance. Women who spoke in village meetings experienced sanctions for disrespect and outspokenness; their husbands were teased for being “controlled.” At endline, participants reported fewer instances of ridicule towards women’s participation. Women expressed a greater sense of solidarity with each other; men reported greater respect for men whose wives contribute. The intervention’s effects appeared more pronounced in areas with greater cultural heterogeneity, suggesting norm change may be harder to affect where norms are tighter.

**Conclusion:** UPWARD provides evidence that gendered social norms change programs can have identifiable impacts on women’s participation in water-related decision-making over a short time. While other interventions have used larger, multi-level strategies to affect gender norms, UPWARD has shown that community mobilization with brief (∼4 months) but concentrated engagement with communities can promote changes in social norms that persist at least 6 months after intervention’s end.

## Introduction

Despite having natural freshwater resources, rural Tanzanians have limited access to safe water and sanitation services. Less than half (42%) of all rural households have access to basic drinking water, while 24% rely on unimproved water sources and a further 20% rely on surface water (World Health Organization and UNICEF, 2020). Water privatization schemes common throughout the country prioritize those most able to pay and may privilege male users as a result, while as those delegated in charge of water-collection and its use in household activities, women and girls bear the unequal labor burden of water scarcity (Brown, 2010). While water fetching, for example, women and girls have increased risk of infection, are exposed to sexual harassment, and face increased risk of injury and experience chronic fatigue from navigating long distances with heavy loads (Caruso et al., 2015).

Key to increasing women and girls’ access to adequate water and sanitation is their ability to participate in water-related decision-making. The involvement of women’s perspectives is central to the provision, management, and safeguarding of water within integrated water resource management (IWRM) (Khosla and Ahmed, 2006). Without this, key water decisions which impact women’s daily lives–such as the cost and placement of water–may be ignored. Moreover, women’s participation in water-related decision making is thought to benefit all water users–for example, by increasing program sustainability through faster repair time of water facilities (Ivens, 2008). Yet evidence from Tanzania shows that even when women sit on village water councils, they have little ability to steer decision-making toward the gendered-nature of water usage or influence final decisions of water councils (Mandara et al., 2017). In Tanzania, inequitable gender norms and limited women’s autonomy has undermined the participation of women within IWRM. Men tend to be primary decision makers regarding family planning (Mosha et al., 2013). Governance patterns and the gendered social norms that drive them prevent women and girls from participating in water-related decisions at the community level.

Efforts to increase women’s participation in governance structures beyond token representation have historically been ineffective. The Government of Tanzania has held quota seats for women for over 30 years, but it is rare for women to be elected to constituency seats, suggesting that gender discrimination against women in institutional positions of power persists despite legislation (Yoon, 2016). The failure of quotas to effect the intended structural change in governance structures is paralleled in other East African countries (Burnet, 2008; Debusscher and Ansoms, 2013; Muriaas et al., 2013; Rosen, 2017; Tinker, 2004). Quotas extend down to village level governance but are often either disregarded or do not translate into legitimate decision-making power. At the time of the study, seven out of 25 seats on Village Government Councils were mandated to be held by women, but several studies have shown that quotas are insufficient as entry points for women to hold meaningful power in decision-making processes related to water (Mandara and Niehof, 2013; Mandara et al., 2017).

Changing the gendered nature of governance in Tanzania is likely to require the changing of gender norms. Most existing gender systems are rigidly hierarchical, reinforcing systematic inequality that undermines the rights of women and girls and sustains inequality in decision-making (Heise et al., 2019). Gender norms are social norms defining acceptable and appropriate actions for women and men in each group or society. They are embedded in formal and informal institutions, nested in the mind, and produced and reproduced through social interaction. They play a role in shaping women and men's (often unequal) access to resources and freedoms, thus affecting their voice, power and sense of self (Cislaghi and Heise, 2020). The adverse health impacts of gendered social norms extend into the WASH sphere. Lack of access to water, sanitation, and hygiene affects women and girls disproportionately and addressing social and gender norms which limit women and girls access to and benefit from WASH interventions is critical to ensuring equitable impact and intervention sustainability (Khosla and Ahmed, 2006).

Social norms theory has been increasingly used throughout low- and middle-income countries to address a variety of health and social development-related challenges (Barker et al., 2010; Linos et al., 2013; Heise and Manji, 2016; Costenbader et al., 2017; Cislaghi, 2018; Cislaghi et al., 2019). The literature on social norms and gender norms is wide and there exist a range of working definitions and concepts used in intervention literature (Bicchieri, 2006; Cislaghi and Heise, 2020). In their broadest sense, social norms are rules of behavior constructed and shared by a group. People follow social norms because they think others follow them and because they believe other people think they should follow them too. Although the exact definition of social norms varies between fields, they have in common the following elements:

• Empirical expectations (EE): What I think others do
• Normative expectations (NE): What I think others expect me to do
• Sanctions: Anticipated opinion or reactions of others
• Referent persons/groups: Individuals (or groups of individuals) whose opinions and reactions matter
• Exceptions: Circumstances under which it is acceptable for someone to break a norm

Intervention efforts to transform gendered social norms continue to evolve our understanding of “what works” to foster change. Critical reflection practices, grounded in popular education approaches, are consistently identified as foundational for shifting gendered norms. Such practices support opportunities to re-consider, rehearse, and internalize evidence and visible actions that support alternate, more equitable norms. For instance, in work to support critical consciousness about masculinities, group activities draw on Freire’s approach to consciousness-raising to support young men to become conscious of current inequitable norms and link their new consciousness to taking alternative actions in support of gender equality (Freire, 1970; Freire, 2000; Kato-Wallace et al., 2019). Such practices require dedicated support to equip facilitators to implement this approach (Bartel, 2018; Jewkes et al., 2020). Evidence also indicates that norms change efforts benefit from working across different levels of the socio-ecological model (e.g., individual, interpersonal, community, societal), and with careful attention to key referent groups, in order to help build a “critical mass” for change (Abramsky et al., 2014; Cislaghi et al., 2019).

“Organized diffusion”—the spread of knowledge through communities and social networks–is a promising framework recently highlighted for its potential to further increase the impact of social norms related programs. By explicitly preparing participants to engage others with their emerging knowledge and skills, such focus may amplify a key pathway by which new ideas spread organically from person to person and community to community–doing so more effectively than broader community mobilization efforts without this specific focus (Cislaghi et al., 2019). This approach runs parallel with key tenets in critical pedagogy, most notably the use of structured critical reflection within and among groups, as a foundational process for shifting gendered power relations (Freire, 1970; Freire, 2000). To date, some form of the organized diffusion approach has shown success in modifying harmful gender norms across a variety contexts, including in Mali, by increasing injunctive norms of female genital cutting (Cissé et al., 2018), in Nepal, by increasing the likelihood of assisting a woman who experienced marital violence (Clark et al., 2020), and in Nigeria, where both direct participants of the Safe Spaces program as well as their closet peers reported favorable changes in attitudes toward gender norms (Welsh et al., 2017). It is also possible that organize diffusion approaches help to avoid common challenges in changing social norms, such as neglecting the influence of indirect social norms or only engineering social norms from the “outside-in” (Cislaghi and Heise, 2018).

The WARIDI project (2016–2021) was a five-year IWRM and WASH project implemented in the Wami-Ruvu and Rufiji river basins in Tanzania. By facilitating government- and community-driven processes, WARIDI sought to increase the use of sustainable, multiple-use water supply systems; strengthen governance for sustainable management of water resources and services; and improve livelihoods through private sector investment opportunities in water services and natural resource management As part WARIDI, a stand-alone, gendered-social norms change (GSNC) activity called Uplifting Women’s Participation in Water-Related-Decision-Making was implemented to increase women’s participation in household and community life within WARIDI water basins areas, with the ultimate goal of improving water resources management.

In September of 2016, WARIDI conducted a gender integration and youth inclusion (GIYI) assessment through 67 key informant interviews to better understand the social norms influencing gendered patterns of labor, representation, and participation in water-related decisions. These social norms were more clearly delineated in formative research in June of 2017 through focus group discussions in Hembeti (Morogoro Region), Lulanzi (Iringa Region) and Kanolo (Morogoro Region). The specific gendered social norms inhibiting women’s water-related decision-making power included:

Expectations that women remain shy and demure, that they do not speak after men or contradict them, and that they lack the confidence and knowledge to participate fully in water-related decisions.
  o For younger women, norms requiring respect and subservience towards elders prevent them from speaking out.
  o Attitudes that youth (in Tanzania, defined as those below 35 years of age) are not responsible for household or village decisions and moreover ignorant of village issues discourages youth attendance and participation in village meetings.
  o Unmarried youth face a particular barrier to participation in governance structures, as they are expected to listen to their parents and rely on them for financial assistance.
  o Married women, who have earned an elevated position due to their marital status, are nonetheless sanctioned for speaking in public because they may reflect poorly on their husbands or give the impression that they control their husbands.
Men’s opinions are regarded as more important and more beneficial to the community; elder men, in particular, are often viewed as final decision makers in village-level meetings
Men are viewed as less in control of their household if their wives appear outspoken in public

Based on the initial gender and youth assessment and formative research, WARIDI designed an intervention to understand how a community-based gendered social norms approach using community facilitation, critical reflection, and organized diffusion, can lead to changes in gendered social norms relating to women's participation in water-related governance structures. UPWARD builds upon global and Tanzania-specific evidence of “what works” to facilitate gendered social norms change.

## Intervention

The program was designed with the following components in mind. First, to harness community mobilization through the principle of organized diffusion (Abramsky et al., 2014; Abramsky et al., 2016; Cislaghi et al., 2019), in which direct participants in an intervention spread ideas across their social network. Second, by focusing on key referent groups, particularly formal leaders (both religious and political), the intervention targets those most likely to influence normative expectations (Bicchieri, 2006). Third, the intervention design used principles of effective facilitator-led sequencing designed to promote critical reflection practices, including motivation (where participants are supported to recognize norms explicitly and reflect on detrimental consequences of norm compliance); deliberation (where participants create a new positive norm within their reference group and identify strategies to motivate others); and action (where participants publicly enact strategies and motivate others to join the group, eventually reaching critical mass of organized diffusion to change norms (Cislaghi, 2018). Cutting across these components, the intervention grounded itself in understanding the intersectional power relations that limit women’s ability to speak in public, and that shape sanctions faced by women and their husbands for transgressing these norms.

**FIGURE 1:**
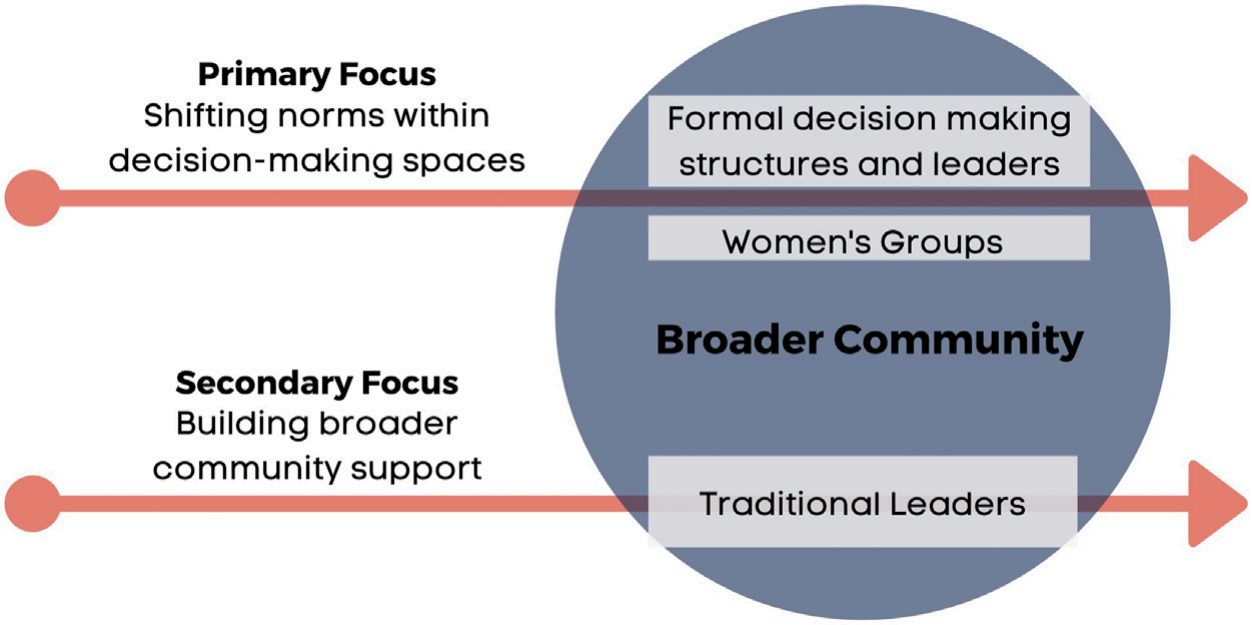
UPWARD intervention focus and target groups.

Two Community Facilitation Teams (CFTs) composed of three women and three men were trained in key gender concepts, identification of social norms impacting WASH roles, facilitating critical reflection about social norms, as well as ways to support women in decision-making, public speaking, and advocacy. Training of the CFTs drew on principles of adult education (Freire, 1970), which emphasizes community dialogue, critical reflection, and collective commitment to action to effect social change. Across four months, the CFTs facilitated a series of UPWARD sessions with two separate audiences. In the first track, CFTs reached elected, religious, and traditional leaders within communities. The leaders track consisted of four sessions, each 2 h in length for a total of 8 h of training time. In the second track, CFTs facilitated sessions with pre-existing women’s groups, primarily Village Community Banking (VICOBA) groups–small, community-based microfinance and lending associations common throughout Tanzania (Figure 1). This design decision was made in order to reach women with some degree of previous leadership and participation experience, as more promising candidates to influence social norm change. The women’s group track varied slightly in Kanolo (16 h total content time) as compared to Lulanzi (14 h of content). Sessions included content related to key gender concepts, the intersection of gender and WASH, social norms change and envisioning the future of the community, models and principles of inclusive leadership, communication skills, and ways to support women’s participation in decision-making.

Applying the principle of organized diffusion and designed using community-led facilitation techniques based in critical reflection approaches, the CFTs delivered the intervention over the course of four months. In total, the intervention directly engaged 229 individuals. In Lulanzi, the intervention reached 36 formal and traditional leaders (24 men, 12 women), and 95 women in women’s groups. In Kanolo, the intervention reached 25 formal and traditional leaders (17 men, eight women) and 73 women in women’s groups. Additionally, the CFTs in both communities conducted a—a community-wide session focused on women’s leadership in water-related decision-making. These sessions, held by community request, reached a total of 164 (65 women, 99 men) community members. The intervention coordinator conducted monthly process monitoring, including implementation fidelity, discussions with and observation of CFTs, and informal interviewing with stakeholders.

## Methods

To evaluate changes in social norms, the intervention used social norms vignettes delivered in focus groups of 6–12 participants (Bicchieri et al., 2014; Stefanik and Hwang, 2017). Vignettes are fictional short stories used to elicit information about social norms as well as personal attitudes, norms, and beliefs. The vignettes were developed based on information gathered through the formative research conducted in May/June 2017 and analyzed using CARE’s Social Norms Analysis Plot (Stefanik and Hwang, 2017). A trained facilitator reads a vignette to participants who are asked to consider how a character will behave in a situation likely to elicit pressure to comply with specific social norms. The fictional situation creates distance between its characters and respondents, better obviating the risk of socially desirable responses (Bicchieri et al., 2014; Gourlay et al., 2014).

UPWARD was evaluated at baseline in December of 2017 and at endline in April 2019. Baseline was conducted in both intervention villages. Endline was conducted in both intervention villages with the addition of two villages, Kiberege and Mtitu, roughly matched on social demographics, to serve as controls. Kanolo and Kiberege are both small towns (∼1,000 residents) in the Kilombero district. Both are predominantly Muslim with minority Christian populations and are ethnically diverse, with the majority of residents Ngindo, Pogoro, Mbunga, Bena, Sukuma, Ndengereko, Yao, Matumba, Sagara, and Nyakyusa. Both towns are also predominantly agricultural. Lulanzi and Mtitu are slightly larger (∼2000 and ∼1,500 residents, respectively), with the vast majority Christians belonging to the Hehe tribe. Agriculture and livestock are the predominant source of income.

FGD participants were grouped by gender and by age (with youth 35 years and younger and elders above 35 years) (Table 1). Across baseline and endline, 366 individuals participated in FGDs using the SNAP, with 103 at baseline and 263 at endline. Individuals were recruited via convenience sampling by either the UPWARD program assistant, WARIDI engagement agent, or the village executive officer (VEO). While detailed background data was not collected from participants, some described direct participation in the intervention (usually women from VICOBAs who attended UPWARD training sessions), but the majority were not. To our knowledge, none of the participants were part of the reference group of traditional or formal leaders. All participants received the background to the study and provided verbal consent to participate.

**TABLE 1:** Participant numbers for the baseline and endline assessments

Focus group discussions	Baseline		Endline			
Group	*Kanolo*	*Lulanzi*	*Kanolo*	*Lulanzi*	*Kiberege*	*Mtitu*
Women <35	12	12	17	10	13	13
Women >35	12	13	6	16	12	12
Men <35	12	13	12	13	10	11
Men >35	16	13	8	10	7	12
Mixed gender <35	—	—	12	8	13	8
Mixed gender >35	—	—	8	11	9	12
Community facilitation teams	—	—	4	5	—	—
Total	52	51	67	73	64	68
In-depth interview participation by village	Baseline		Endline			
Women <35	3	3	0	1	1	2
Women >35	8	8	5	5	4	3
Men <35	4	4	4	0	1	1
Men >35	7	7	1	5	4	4
Total	22	22	10	11	10	10

UPWARD used two vignettes, the same at baseline and endline (Table 2). The program also conducted in-depth interviews across stakeholder groups with a semi-structured interview script, assessing village meeting procedures, women’s participation in community meetings, and referent individuals within the community.

**TABLE 2:** Social norm vignettes.

	Vignette summary	Gendered social norms prompted in the vignette
Vignette 1	Faraja, an unmarried woman of 22, is considering if she should participate in a village meeting. She wants to impress on the community the importance of paying water bills. When she finally speaks, her voice is low and hard to hear	• Women should be shy and demure
	—	• Women should not speak after or contradict men
	—	• Women lack confidence/knowledge to participate in civic life
Vignette 2	Salma, a married woman age 28, advocates in a village meeting for a new water point that is more advantageous to women. Other men in the community speak to her husband, Rashid, age 31, about her participation. Rashid later speaks to Salma at home	• Married women should defer to their husbands; men should have control over their wives
	—	• Elder men make the final decisions for the village on behalf of women

### Evaluation and the Social Norms Analysis Plot

Evaluation methodology drew heavily on the Social Norms Analysis Plot (SNAP), a tool piloted by CARE International and developed for use in resource-constrained settings (Stefanik and Hwang, 2017). Briefly, a social norm exists and applies to a specific situation when the empirical and normative expectations of different groups are mutually consistent (Bicchieri, 2006). Areas in which there is disagreement between groups and between time periods suggest changes in social norms. FGD responses to vignettes were analyzed, coded, and compared to other participant groups, to the baseline evaluation, and to the intervention-naïve “control” communities. The analysis and choice of themes at endline was based on the following questions:

• Are there indications that disagreement is increasing about EE and/or NE among certain groups, and if so, why?
• Are social sanctions lessening or weakening over time?
• Are more alternative, non-normative behaviors perceived to be possible, or occurring?
• Are there changes in the conditions in which it is acceptable to deviate from the norm?

Data was coded using inductive methods and thematic analysis. Emergent themes were verified with the field research team. Analysis included magnitude coding, which can be used to identify intensity, frequency, and direction of qualitative content, and is particularly useful when comparing intervention and control sites (Saldana, 2014).

## Results

### Baseline

At baseline in both communities, participants described how uncommon it is for women (particularly younger women) to express themselves in village meetings. Participants noted it is uncommon for men to take seriously women’s participation in decision-making structures. Men and women described scornful attitudes towards women who speak out in village meetings. Reacting to Faraja’s advocacy for water payment in the first vignette, one woman described how regularly men harass women during meetings: “They may start shouting while women speak and thus will frighten her.” Some participants implicitly tied women’s demonstration of timidity in public spheres to the normative expectations of a wife’s behavior at home. About Faraja expressing ideas in a village meeting, a female participant noted that “Women themselves may disapprove … They may say, ‘If a young woman is speaking like this now, what will happen when she gets married? She will control her husband.’”

There was some suggestion of divergent expectations around unmarried and married women’s role in the public sphere. While there was still a general concurrence that women’s opinions are valued lower than men’s opinions, many more participants viewed it as acceptable that Salma, a married women, would speak in village meetings. By tying the acceptability of participation to marriage status, social norms reinforce the belief that men’s voices are to be held in higher regard. At the same time, participants expressed conflicting ideas about the role marriage plays in decision-making power within the community. In addition to sanctions against women for speaking (e.g., jeering and name-calling), men may also be expected to “control” their wives by limiting their participation in the public sphere. Participants jokingly referred to men who “had no control over their wives” as *bushoke,* disparagingly referencing a popular song in which a man is forced by his wife to sleep on their floor (Reuster-Jahn and Kießling, 2006).

A final theme was a perceived “higher bar” for women to reach in order for their opinions to be heard and considered. While some participants expressed the belief that participation from men and women was valued equally, others were more forthcoming. As one older male participant explained during the vignette featuring Salma, “In our custom even if what a woman speaks is good, it has to be said by a man and not woman.” In Lulanzi, a common theme repeated by participants in interviews was that it did not matter if a woman spoke, so long as what she said was a “good contribution” and benefitted the community–otherwise she risked the scorn of fellow women as well as men. Men, whose wives were perceived to be “talkative” during meetings without adding valuable contributions, were also derided by their male peers as ones who could not control their wives. Although the same expectations may also apply to men–as suggested by more general statements, e.g. “it doesn’t matter who speaks so long as what is said is for the good of the community,” there may be higher expectations for women's opinions to be well spoken and beneficial for the larger community.

### Endline

While many of the baseline findings held true following the intervention, there were numerous indications of possible shifts in social norms within and between groups. When assessing changes between baseline and endline, we paid particular attention to both differences between the two time points, as well as to changes in the extent of conformity and disagreement between and within groups, following the guidance lade out by CARE International (Stefanik and Hwang, 2017). More detailed findings are summarized in Table 3.

**TABLE 3:** Social norms analysis plot.

Norm components	Definition	Indicators of change	Lulanzi	Kanolo
Empirical expectations	What I think others do	• responses reflect a different perception of what people think others are doing	• more women expressing the expectation that men would support wives for participating in village meetings	• increase in respondents reporting women speaking out in village meetings
		• increase in respondents report a perceived change of behavior of others	• less expectation of ridicule for women participating in village meetings	• changes more notable in elder participants (who were primary beneficiaries of UPWARD trainings) and in young men compared to young women (possibly because young men more often attend village meetings)
		• changes in the extent of conformity and disagreement among homogenous groups, and across the different groups	• greater expectation that men participate in household activities	• increase in women reporting seeing men engaging in household chores
		—	—	• increase in expectations that village leaders will encourage women’s participation
		—	—	• fewer reports of nagging or ridicule at women speaking out
Normative expectations (NE)	What I think others expect me to do (what I should do according to others)	• responses reflect a different perception of what others expect respondents to do	• more women believed public participation to be their right, however this change in normative expectations only extended to how they expected other women to respond to their participation—not men	• more men reporting that it is expected of them to participate in household activities such as fetching water and childcare
		• increase in respondents reporting the desired new behavior as expected of them	• some women described the difficulty of changing their normative expectations, mainly when they have not seen other women participating: "We have grown up seeing only men speak in village meetings. Therefore, it is stuck in our mind that this is how it is supposed to be"	• greater indication that women, especially women over the age of 35, feel that both other women and men expect and want them to participate more in village meetings
		• changes in the extent of conformity and disagreement among homogenous groups, and across different groups	—	• "she should try to speak out by herself because no one can deliver the message like her"—Youth male group
		• changes in alignment between empirical and normative expectations	—	• increasing disagreement between elder groups (greater expectations for women’s participation) and younger groups (similar findings to baseline)
Sanctions	Anticipated opinion or reaction of others	• changes in the sanctions that are identified	• compared to baseline, fewer sanctions (from other men) against a man whose wife has argued for a point in a village meeting	• many FGDs showed reductions in social sanctions for a woman advocating a position in a village meeting
		• changes in the severity of sanctions	—	o More women would support and congratulate Faraja for speaking out
		• changes in the likelihood of sanctions being enacted	—	o Fewer men would insult rashid for Salma’s participation in the village meeting
		• changes in consistency across groups	—	o More respondents expected that rashid would support and congratulate Salma, rather than demean her for participating
		—	—	• some indication of sanctions for men who disapprove of women speaking in village meetings
Sensitivity to sanctions	Do sanctions matter for behavior?	• changes in how the main character would respond to negative sanctions	• some indications that women experience fewer sanctions in public speaking, but only if their opinions benefit the whole community	• more respondents, both men and women, expecting female vignette characters to continue making their point despite booing or other signals of disapproval
		• increase in respondents who say the main character would still behave in the desired way despite sanctions	—	• some participants expressed how rashid, after experiencing criticism from other men for being "controlled" by his wife, would push back and educate his peers on the importance of women's participation in village life
		—	—	• elder men, in particular, showed the most significant changes in support. As one man explained, “rashid will tell his wife to continue to stand and fight for what she believes is right for their village and not to think about what her fellow villagers would say.”
Exceptions	Under what circumstances would it be okay for the main character to break the norm?	• change in the # of exceptions allowed to break a norm	• more men recalling examples of women whose suggestions in village meetings have been adopted by the community	• compared to baseline, less discussion about how a woman's contribution must be a "good" suggestion to be taken into consideration; more men, in particular, reported the importance of women's views
		• changes in # or types of individuals who deviate from the norm	—	• increases in the number of individuals who report that women freely speak out in meetings
		• changes in responses about individuals who are impervious to social sanctions	—	• widowed women, in particular, expressed less fear of sanctions in village meetings, likely because they face less pressure and fear of repercussions from their husband at home

A higher proportion of men compared to baseline regarded women’s participation (and specifically that of their wives) in village meetings as beneficial to the community as a whole, particularly when it comes to water-related knowledge. FGDs suggested two reasons for this shift. First, normative expectations among men have changed. In the baseline, many men believed that a man whose wife voiced her opinion in a village meeting would be ridiculed by other men for being “controlled” by his wife. Although this expectation remained present at endline, it was far less prominent. Instead, a greater number of men regarded having an outspoken wife as beneficial to both the community and their own standing among other men. Both men and women anticipated that a man with an outspoken wife would be complimented as often as he would be ridiculed.

Young men in Kanolo explained that other men would congratulate Rashid when Salma makes her case in the community meeting, telling him, “he has a strong woman who can stand and contribute her opinions.” In Kanolo, when one elder woman stated that men will say Rashid has been “bewitched,” another countered: “These days, men like Rashid will take this as a positive change. He may even tell them, ‘If she bewitches me, then she loves me; therefore, I have no problem with that.’” One participant in the mixed youth focus group explained that even if one of Rashid’s friends insulted him, “Rashid would try to educate his friend about the importance of giving their wives a right to speak their opinions, both at the family level and in village meetings.” There was also some indication that men who disapprove of women’s participation now experience sanctions from the community which previously did not exist. One example included a man who was reprimanded by other community members when interrupting a woman talking. An elder woman in Kanolo stated, “Women know their rights, and you can't just drag them around,” while discussing how women now supported each other more in meetings.

Male youth in particular expressed stronger convictions on the importance of women’s participation in water-related decision-making structures. One participant in the youth male group in Kanolo argued that Faraja “should try to speak out by herself because no one can deliver the message like her.” However, Faraja’s age was also commonly cited as a barrier to her participation, suggesting the deeper sanction that young and particularly unmarried women face for meeting participation. This dynamic was also reflected in youth FGDs, which required greater efforts from facilitators to elicit responses, particularly among younger women. In general, younger women, who the intervention did not directly reach (and by extension, whose reference groups were less likely to experience any diffusion) continued to expect substantial sanctions for participating in village meetings. When asked to provide examples of women who might support Faraja in meeting, younger women cited only older women in the village. Similarly, younger women generally expressed fear of reprisal from husbands: “Sometimes [men] even warn us from home that they do not want to be disgraced in the meetings, therefore we should keep quiet.”

Fewer men, however, expressed the expectation that other men would insult or ridicule Rashid when Salma, a married woman, participates in the village meeting. Indeed, more respondents expected that Rashid would support and congratulate Salma. As one elder woman in Kanolo explained, “Men now expect women to stand up and speak their views.” Elder men in Kanolo explicitly tied this to awareness raising from UPWARD sessions, explaining that they agreed with Salma’s suggestion to change water points as they are aware how a different location would reduce the negative impacts on women and children that come from their burden fetching water.

Finally, there was some indication in Kanolo of shifts in traditionally gendered behaviors. Members of the CFT, interviewed as part of the endline, estimated from their observations in their respective villages that changes stemming from UPWARD sessions had impacted roughly half of the community; men no longer felt embarrassed about fetching water, carrying children, or cleaning the house, and women's workload had been reduced as a result. One older woman reported in an FGD: “In the past, you would have seen a family coming from farming, a man walking majestically at the front with empty hands, while a woman is behind, pregnant, carrying hoes and firewood. However, that has somehow changed.”

Indeed, men explained that it was now expected of them to participate in household activities–fetching water and childcare. As one female respondent in an IDI explained of a couple who live near her: “The man used to be very arrogant and would say if a woman wants me to fetch water, she should also pay for the dowry. However, it is interesting to see him fetching water these days.” Others spoke of men taking on more of a role in everyday household activities, including childcare. This change in attitudes toward men’s roles was echoed in reflections from the male members of the Kanolo CFT, one of whom described at first feeling embarrassed to engage in activities traditionally gendered as female (e.g., water-fetching), but later finding that his entire household worked more comfortably together because of this. However, no men in the FGDs discussed similarly engaging in household chores. To what extent men engaged in different activities and why (particularly whether they engaged in new activities because of viewing them as “right” vs. feeling they might experience sanctions if they did not) remains an open question.

Despite positive changes, there were still many indications across every age group and within each village that harmful gender norms persist. The endline evaluation indicated that modifications in empirical expectations and normative expectations were substantially less in Lulanzi. Responses to FGD vignettes indicate that though women’s group members felt confident in their own opinions, they feared expressing these opinions because their empirical expectations regarding what happens when women speak out had not changed. As an elder woman in Lulanzi explained: “We have grown up seeing only men speak in village meetings. Therefore, it is stuck in our mind that this is how it is supposed to be.”

Men in Lulanzi indeed continued to express harmful normative beliefs. When, in one of the vignettes, Salma advocates for the placement of a water point that is more advantageous to women, some male participants thought that, even if Salma raised a valid and useful point, she would have “disrespected men” by speaking. Participants across age and gender groups were quick to cite the strength of patriarchal social norms in Lulanzi and, compared to Kanolo, there were few comments on changes in men’s attitudes or behavior. These findings point to the importance of engaging men and boys in self-reflection and as agents of change within gendered social systems, as has been done, for instance, in microfinance programs (Pawlak et al., 2012).

#### Control Villages

In general, findings in Kiberege, where no UPWARD intervention took place, appeared similar to the baseline findings in Kanolo. Compared to Kanolo, participants in Kiberege held low empirical expectations for how many women participate in village meetings. When participants were asked, “Out of 10 women in the village, how many do you think would support Faraja speaking?” responses in Kanolo averaged around 5. They ranged from two among young women, five in both elder women and young men, and seven in elder men. In contrast, nearly every group in Kiberege expected no more than one or two men to support Faraja.

We also asked participants “Out of 10 men in the community, how many would disapprove of Salma speaking up in the meeting?” In Kanolo, responses were low: between one and three men. In Kiberege, they ranged from 5 to 8. Men in Kanolo tended to think that other men would support Salma’s participation and compliment Rashid on the fact he had married Salma. In Kiberege, results were mixed. Men in FGDs thought that only a few men would speak positively about Salma’s participation to Rashid. In the mixed elder FGD, some thought that Rashid would ask his wife not to speak. Female youth thought that only a small percentage of men in the community would support their wife speaking up; otherwise, peer shaming by other men is common. As one participant in the mixed gender youth session stated: “husbands like Rashid are very few in Kiberege.”

Comparisons between Lulanzi and Mtitu are made difficult by the fact that, unknown to the research team before choosing the site as a control, the USAID Nafaka (part of Feed the Future) program had conducted women’s empowerment training for men and women, focusing increasing the number of women in capacity-building training, and promoted greater participation of women in leadership positions (Agrilinks Team, 2019). In general, respondents in Mtitu held more favorable expectations regarding women’s participation and there were fewer reports of women experiencing sanctions for participating. Despite this, there were some notable differences between the communities which point to the specific impact of UPWARD.

One sign of the impact of UPWARD training was in women’s empirical expectations regarding women’s support for female participation. Women in Lulanzi were more likely than women in Mtitu to hold expectations that other women would support female participation. Conversely, men’s support for women’s participation was lower in Lulanzi compared to Mtitu. In the vignettes, we asked participants out of 10 men in the community, how many would disapprove of Salma speaking up in the meeting? In Lulanzi, responses were mostly high–ranging from 2 (elder males) to 9 (younger women) and between 5-8 in all other groups. Responses in Mtitu were much lower—four in the eldest male group, and between 2-3 in all other groups. Together, these two examples provide some insight into the successes and shortcomings of the UPWARD program. Although the Lulanzi CFT provided training to male leaders in the village council or the church, the majority of their training occurred in pre-existing women's groups, and thus women were the primary beneficiaries. In the group interview, the CFT reported that they received significant resistance from men regarding some of the gender training, particularly in sessions which focused on the differences between gender and sex. It appeared to be a commonly held belief among men in Lulanzi that women’s low levels of participation were due to “women’s nature,” rather than social norms, particularly the fear of sanctions among women. In Mtitu, in contrast, gender training from the USAID Nafaka program was delivered jointly to men and women. It is possible that higher reach with men has contributed to the discrepancy in men between Lulanzi and Mtitu.

## Discussion

The UPWARD intervention shows that community-facilitated gender norms programming using organized diffusion can have meaningful changes on women’s participation in decision-making structures over a short time period and with limited resources. Over the course of ten months, CFTs, through targeted sessions with community leaders and women’s groups were able to cultivate greater support for women’s perspectives in water-related decision-making; a stronger sense of solidarity among women directly participating in the program; a larger number of men actively encouraging women’s participation in village life and less sanctioning for men whose wives participate; and some indication of men adopting household activities typically regarded as within the women’s domain. As a stand-alone activity in a large-scale WASH project, UPWARD’s success offers a blueprint in both future WASH programming and development more generally.

The SASA! Intervention in Uganda (on which UPWARD drew to adapt facilitated exercises supporting participants to reflect on interlocking normative expectations across different actors in the community at household, peer, and leadership levels) demonstrated community impacts on intimate partner violence at the community level (Abramsky et al., 2014). Cislaghi et al. (2019), in an evaluation of three community-based interventions relying on the principle of organized diffusion, similarly found that participants can be effectively empowered to share their new knowledge and understandings widely within networks, leading over time to changes in social norms.

There is also a variety of norms change literature within the WASH sphere, particularly at the intersection of community led total sanitation, open defecation (OD), and social norms. The UNICEF Pakistan WASH Sustainability Check investigated OD-free status in rural areas of Pakistan. That evaluation found that a weak point in the social norms programming was a low belief in sanctions surrounding OD, resulting in low latrine use rates and low empirical expectations regarding others’ latrine use (Ministry of Climate Change Government of Pakistan, 2016). In contrast, in an evaluation of an OD free campaign in Tamil Nadu, women were more likely than men to expect social sanctions when deviating from what is perceived as prevalent behavior, resulting in greater psychosocial stress (Kuang et al., 2020). In Vietnam, changes in gendered social norms within a WASH program were attributed to increased information and knowledge among women, allowing them to have a voice in technical decision-making within households, although this does not translate more widely into the public sphere (Leahy et al., 2017).

Our findings also illustrate the importance of an intersectional lens to axes of advantage and disadvantage experienced by women. In particular, marriage acts as both a protective and limiting factor. Our findings showed that married women are granted greater respect and authority than unmarried women within the community, while widowed women also experience fewer sanctions for community participation. However, for widows, greater ease in participation may be balanced by a loss of financial autonomy, if they also lose access to income or assets. Widows and divorcees are disadvantaged, for instance, in the adoption of adaptive climate strategies and water management (Van Aelst and Holvoet, 2016). There was also indication that some widowed women were better able to act as trendsetters–initiators of norm abandonment (Bicchieri and Funcke, 2018). While not an explicit goal of the intervention, this approach offers an avenue for future research.

There are numerous lessons for future interventions and for social norms programming Because the intervention targeted women's groups, its direct reach to youth was low. This fact reflected in FGDs, where younger participants were more likely to expect women like Faraja to stay quiet or to imagine harsher sanctions for women's participation. Low participation in village meetings among younger participants also meant that empirical expectations (and thus normative expectations) remain little changed for youth.

Our evaluation points towards fear of sanctions remaining a perhaps insurmountable barrier for interventions failing to more explicitly target men within households, rather than just community leaders. It remains difficult for women to voice their opinions both in village meetings and in the household. Many women reported that despite a shift in personal normative beliefs (e.g. from *It is not my place to participate in meetings* to *I should participate in village meetings*)*,* the fear of sanctions, particularly from their husbands, inhibits their ability to transgress social norms around women’s role in the public sphere. In other words, even if the intervention was successful in altering personal normative beliefs, it was insufficient in modifying women’s normative expectations–their beliefs about others beliefs–and in particular the sanctions they expect men to enforce. All of this suggests the necessity of involving men more directly in future organized diffusion approaches. Individual level male engagement interventions, such as the Bandebereho couples intervention in Rwanda, have shown significant decreases in intimate partner violence (Doyle et al., 2018), although that intervention also showed men continuing to dominate household decision-making. Further research is needed to explore the synergistic and possibly multiplicative impacts of combining male engagement with organized diffusion strategies.

Another possible explanation for the relative strength of the intervention in Kanolo as compared to Lulanzi lies in the social make-up of each village. We suggest gendered social norms may be stronger in villages in which there is high cultural homogeneity. A reference network is composed of people who matter to an individual’s choices; behaviors and beliefs of people inside a reference network matter to behavior, while those outside matter little or not at all (Bicchieri and Funcke, 2018). In Kanolo, participants explained that some shifts in gender norms were due to the more intertribal nature of village life. Greater migration from other areas of Tanzania and the melting pot of cultural norms specific to each tribe have meant that norms may be more easily weakened because of more considerable heterogeneity in empirical expectations and normative expectations. While our research was not explicitly designed to evaluate this finding, it suggests opportunities for future research.

Transgressing a norm can be costly due to sanctions imposed on individuals. It is common to observe a slow change in empirical expectations as people observe few initial transgressors of the norm. Trendsetters are those individuals who may be less sensitive to the sanctions for transgressing a norm. Many of the women interviewed in Kanolo who had taken part in UPWARD training were widowed. Because these women did not have husbands to exert control over their behavior, they had greater autonomy in their decision-making. As one woman explained, "Since I am widowed, … no one will ever mistreat me." Further research would be needed to explore the relative efficacy and potential unintended consequences of targeting widowed women as “first movers” willing to spark change.

Conversely, our findings also suggested an even more substantial barrier faced by younger women, particularly unmarried women, in participating in village governance structures. Women under age 35 were more likely than women over 35 to predict that the women characters in the vignette would not participate due to sanctions or to agree with men due to fear of “disrespect.” Even within youth female FGDs, the project’s female facilitators expressed difficulty in eliciting responses. This so-called “shyness” was similarly mirrored in mixed-gender FGDs, in which few female participants felt comfortable speaking in front of their male peers.

Our findings also suggest that more explicitly targeting men in group settings or in mixed gender settings would be more impactful. Fear of sanctions, particularly from men, was a common reason that women gave when asked why they did not participate in village meetings despite desiring to. The lack of perceived sanctions in Mtitu suggests that USAID Nafaka’s gender training had some success in lessening men’s exclusion of women in village governance. Analyses in other contexts show women may be more likely to expect social sanctions if they deviate from perceived normative expectations, as an analysis of an OD campaign in Tamil Nadu showed (Kuang et al., 2020), while other interventions which have taken gender-transformative approaches have had success in targeting men (Doyle et al., 2018).

Our study has limitations. First, while we followed established methodology (Stefanik and Hwang, 2017) in analyzing vignette FGDs, there are inherent limitations in qualitative analysis. Because we did not record FGDs to ensure participants felt fully comfortable expressing their ideas, we are not able to quantitatively assess, for example, changes in the number of times specific norms or sanctions were mentioned. However, at least one facilitator for both men’s and women’s groups was the same at both baseline and endline, and the extensive notes taken during the session provides further support for the changes described above. We were also unable to conduct individual surveys assessing social norms, which would have provided data which would more allow more accurate comparison between time points. With relatively limited resources, we were also unable to more accurately quantify how using organized diffusion approaches impacted the intervention.

Another limitation lies in the perspectives we were able to hear. Because of the FGD recruiting process and the difficulties of accessing participants during farming season, there were fewer youth participants. While demographic data was not available for the communities, anecdotal reports from participants suggest that many youth leave smaller villages for work. There is also the possibility that by targeting VICOBAs, groups whose aim is implicitly aligned with women’s empowerment, the intervention reached members of the community already primed to advocate for women’s roles in community decision-making structures. However, because the evaluation sampled randomly from the community and did not explicitly target beneficiaries, this could also be considered a strength. Further strengths include the use of vignettes, which limit the evaluation’s susceptibility to socially desirable responses. UPWARD was also independently evaluated by USAID’s Passages project, with similar findings (Passages Project, 2019). The End of Project Evaluation found that the changes in social norms described at endline remained (if not gaining strength), one and a half years following the endline evaluation (George et al., 2020).

The UPWARD intervention has provided evidence that GSNC programs can have identifiable impacts on the acceptance of women’s participation in water-related decision-making over a short period of time (i.e. 4 months of active engagement), albeit with additional dedicated time and resources required for formative research and intervention development and planning, as well as ongoing engagement within the larger project. While other GSNC interventions have used larger, multi-level strategies to affect gender norms, UPWARD has shown that targeted community mobilization requiring relatively limited resources can nonetheless promote greater acceptance of women’s participation in water-related decision-making structures, as well as signs of intermediate changes in social norms. Given the nature of social norms to shift only slowly, it is possible that UPWARD’s full impact on women’s water-related decision-making will continue to evolve.

## Data Availability

The original contributions presented in the study are included in the article/Supplementary Material, further inquiries can be directed to the corresponding author.

## References

[B1] Abramsky T. Devries K. Kiss L. Nakuti J. Kyegombe N. Starmann E. (2014). Findings from the SASA! Study: A Cluster Randomized Controlled Trial to Assess the Impact of a Community Mobilization Intervention to Prevent Violence against Women and Reduce HIV Risk in Kampala, Uganda. BMC Med. 12, 122. 10.1186/s12916-014-0122-5 25248996 PMC4243194

[B2] Abramsky T. Devries K. M. Michau L. Nakuti J. Musuya T. Kyegombe N. (2016). The Impact of SASA!, a Community Mobilisation Intervention, on Women's Experiences of Intimate Partner Violence: Secondary Findings from a Cluster Randomised Trial in Kampala, Uganda. J. Epidemiol. Community Health 70 (8), 818–825. 10.1136/jech-2015-206665 26873948 PMC4975800

[B3] Agrilinks Team (2019). Time Diaries: How a Methodology Change Empowered a Community and Sparked the Fight against GBV. Feed the Future: Agrilinks Available at: https://www.agrilinks.org/post/time-diaries-how-methodology-change-empowered-community-and-sparked-fight-against-gbv. (Accessed March 01, 2021).

[B4] Barker G. Ricardo C. Nascimento M. Olukoya A. Santos C. (2010). Questioning Gender Norms with Men to Improve Health Outcomes: Evidence of Impact. Glob. Public Health 5 (5), 539–553. 10.1080/17441690902942464 19513910

[B5] Bartel D. (2018). Training And Mentoring Community Facilitators To Lead Critical Reflection Groups For Preventing Vioence against Women. The Prevention Collaborative. 10.14361/9783839445860

[B6] Bicchieri C. Funcke A. (2018). Norm Change: Trendsetters and Social Structure. Soc. Res. Int. Q. 85 (1), 1–21.

[B7] Bicchieri C. Lindemans J. W. Jiang T. (2014). A Structured Approach to a Diagnostic of Collective Practices. Front. Psychol. 5, 1418. 10.3389/fpsyg.2014.01418 25538666 PMC4257103

[B8] Bicchieri C. (2006). The Grammary of Society: The Nature and Dynamics of Social Norms. Baltimore: Cambridge University Press.

[B9] Brown R. (2010). Unequal burden: Water Privatisation and Women's Human Rights in Tanzania. Gend. Develop. 18 (1), 59–67. 10.1080/13552071003600042

[B10] Burnet J. E. (2008). Gender Balance and the Meanings of Women in Governance in Post-Genocide Rwanda. Afr. Aff. 107 (428), 361–386. 10.1093/afraf/adn024

[B11] Caruso B. A. Sevilimedu V. Fung I. C.-H. Patkar A. Baker K. K. (2015). Gender Disparities in Water, Sanitation, and Global Health. The Lancet 386 (9994), 650–651. 10.1016/S0140-6736(15)61497-0 26334153

[B12] Cislaghi B. Denny E. K. Cissé M. Gueye P. Shrestha B. Shrestha P. N. (2019). Changing Social Norms: The Importance of “Organized Diffusion” for Scaling up Community Health Promotion and Women Empowerment Interventions. Prev. Sci. 20, 936–946. 10.1007/s11121-019-00998-3 30747395 PMC6647388

[B13] Cislaghi B. Heise L. (2020). Gender Norms and Social Norms: Differences, Similarities and Why They Matter in Prevention Science. Sociol. Health Illn 42 (2), 407–422. 10.1111/1467-9566.13008 31833073 PMC7028109

[B14] Cislaghi B. Heise L. (2018). Theory and Practice of Social Norms Interventions: Eight Common Pitfalls. Glob. Health 14 (1), 7. 10.1186/s12992-018-0398-x PMC6098623 30119638

[B15] Cislaghi B. (2018). The story of the "Now-Women": Changing Gender Norms in Rural West Africa. Develop. Pract. 28 (2), 257–268. 10.1080/09614524.2018.1420139

[B16] Cissé M. Gueye P. Manel V. (2018). A Community-Led Approach to Community Empowerment in Mali. Mauritania, Guinea, and Guinea-Bissau: Tostan. [Endline evaluation brief].

[B17] Clark C. J. Shrestha B. Ferguson G. Shrestha P. N. Calvert C. Gupta J. (2020). Impact of the Change Starts at Home Trial on Women's Experience of Intimate Partner Violence in Nepal. SSM - Popul. Health 10, 100530. 10.1016/j.ssmph.2019.100530 31890850 PMC6928358

[B18] Costenbader E. Lenzi R. Hershow R. B. Ashburn K. McCarraher D. R. (2017). Measurement of Social Norms Affecting Modern Contraceptive Use: A Literature Review. Stud. Fam. Plann. 48 (4), 377–389. 10.1111/sifp.12040 29165824

[B19] Debusscher P. Ansoms A. (2013). Gender Equality Policies in Rwanda: Public Relations or Real Transformations? Develop. Change 44 (5), 1111–1134. 10.1111/dech.12052

[B20] Doyle K. Levtov R. G. Barker G. Bastian G. G. Bingenheimer J. B. Kazimbaya S. (2018). Gender-transformative Bandebereho Couples' Intervention to Promote Male Engagement in Reproductive and Maternal Health and Violence Prevention in Rwanda: Findings from a Randomized Controlled Trial. PLOS ONE 13 (4), e0192756. 10.1371/journal.pone.0192756 29617375 PMC5884496

[B21] Freire P. (1970). Pedagogy of the Oppressed. 30th Anniversary Edition. New York: Seabury Press.

[B22] Freire P. (2000). *Pedagogy Of the Oppressed*. Continuum. New York: Bloomsbury.

[B23] George J. Sudi C. Logan M. Kinyage P. Marandu R. (2020). End of Project Gender Integration and Youth Inclusion Learning Research. Morogoro: USAID/Tanzania Water Resources Integration Development Initiative.

[B24] Gourlay A. Mshana G. Birdthistle I. Bulugu G. Zaba B. Urassa M. (2014). Using Vignettes in Qualitative Research to Explore Barriers and Facilitating Factors to the Uptake of Prevention of Mother-To-Child Transmission Services in Rural Tanzania: A Critical Analysis. BMC Med. Res. Methodol. 14 (1), 21. 10.1186/1471-2288-14-21 24512206 PMC3922981

[B25] Heise L. Greene M. E. Opper N. Stavropoulou M. Harper C. Nascimento M. (2019). Gender Inequality and Restrictive Gender Norms: Framing the Challenges to Health. The Lancet 393 (10189), 2440–2454. 10.1016/S0140-6736(19)30652-X 31155275

[B26] Heise L. Manji K. (2016). *Social Norms* (Professional Development Reading Pack No. 31). Birminham: GSDRC.

[B27] Ivens S. (2008). Does Increased Water Access Empower Women? Development 51 (1), 63–67. 10.1057/palgrave.development.1100458

[B28] Jewkes R. Willan S. Heise L. Washington L. Shai N. Kerr-Wilson A. (2020). *Effective Design and Implementation Elements in Interventions to Prevent Violence against Women and Girls* [Global Programme Synthesis Product Series]. Pretoria: South African Medical Research Council Available at: https://www.whatworks.co.za/documents/publications/373-intervention-report19-02-20/file. (Accessed March 25, 2020).

[B29] Kato-Wallace J. Barker G. Garg A. Feliz N. Levack A. Ports K. (2019). Adapting a Global Gender-Transformative Violence Prevention Program for the U.S. Community-Based Setting for Work with Young Men. Glob. Soc. Welf 6 (2), 121–130. 10.1007/s40609-018-00135-y 30956935 PMC6444362

[B30] Khosla P. Ahmed S. (2006). Resouce Guide: Mainstraming Gender in Water Management. Harare: Gender and Water AllianceUNDP. 10.1142/9789812772381_0063

[B31] Kuang J. Ashraf S. Shpenev A. Delea M. G. Das U. Bicchieri C. (2020). Women Are More Likely to Expect Social Sanctions for Open Defecation: Evidence from Tamil Nadu India. PLOS ONE 15 (10), e0240477. 10.1371/journal.pone.0240477 33048969 PMC7553302

[B32] Leahy C. Winterford K. Nghiem T. Kelleher J. Leong L. Willetts J. (2017). Transforming Gender Relations through Water, Sanitation, and hygiene Programming and Monitoring in Vietnam. Gend. Develop. 25 (2), 283–301. 10.1080/13552074.2017.1331530

[B33] Linos N. Slopen N. Subramanian S. V. Berkman L. Kawachi I. (2013). Influence of Community Social Norms on Spousal Violence: A Population-Based Multilevel Study of Nigerian Women. Am. J. Public Health 103 (1), 148–155. 10.2105/AJPH.2012.300829 23153124 PMC3518349

[B34] Mandara C. G. Niehof A. (2013). Does Women’s Representation in Local Water Management Lead to Better Meeting Women’s Domestic Water Needs? Int. J. Soc Sci. Humanity Stud 5 (1), 210602.

[B35] Mandara C. G. Niehof A. van der Horst H. (2017). Women and Rural Water Management: Token Representatives or Paving the Way to Power? Water Alternatives 10 (1), 18.

[B36] Ministry of Climate Change Government of Pakistan (2016). Sector Sustainability Check Study: Rural Open Defecation Free (ODF) & Rural (Drinking) Water Supply Schemes (RWSS). Islamabad: Itad - London.

[B37] Mosha I. Ruben R. Kakoko D. (2013). Family Planning Decisions, Perceptions and Gender Dynamics Among Couples in Mwanza, Tanzania: A Qualitative Study. BMC Public Health 13 (1), 523. 10.1186/1471-2458-13-523 23721196 PMC3679800

[B38] Muriaas R. L. Tønnessen L. Wang V. (2013). Exploring the Relationship between Democratization and Quota Policies in Africa. Women's Stud. Int. Forum 41, 89–93. 10.1016/j.wsif.2013.05.010

[B39] Passages Project (2019). *Case Study: Uplifting Women’s Participation In Water-Related Decision-Making Project* (Norms across Sectors Case Study: WASH). Washingotn, DC: USAID Passages.

[B40] Pawlak P. Slegh H. Barker G. (2012). Journeys of Transformation: A Training Manual for Engaging Men as Allies in Women’s Economic Empowerment. CARE Int. Promundo, 1–88. 10.21236/ada572948

[B41] Reuster-Jahn U. Kießling R. (2006). *Lughya Ya Mitaani In Tanzania: The Poetics And Sociology Of a Young Urban Style Of Speaking* (No. 13; Swahili Forum). Mainz: Department of Anthropology and African Studies, Johannes Gutenberg University.

[B42] Rosen J. (2017). Gender Quotas for Women in National Politics: A Comparative Analysis across Development Thresholds. Soc. Sci. Res. 66, 82–101. 10.1016/j.ssresearch.2017.01.008 28705365

[B43] Saldana J. (2014). The Coding Manual for Qualitative Researchers. 3rd ed. Newbury Park: SAGE Publications Ltd.

[B44] Stefanik L. Hwang T. (2017). Applying Theory to Practice: CARE’s Journey Piloting Social Norms Measures for Gender Programming. Atlanta: Cooperate for Assitance and Relief Everywhere (CARE).

[B45] Tinker I. (2004). Quotas for Women in Elected Legislatures: Do They Really Empower Women? Women's Stud. Int. Forum 27 (5–6), 531–546. 10.1016/j.wsif.2004.09.008

[B46] Van Aelst K. Holvoet N. (2016). Intersections of Gender and Marital Status in Accessing Climate Change Adaptation: Evidence from Rural Tanzania. World Develop. 79, 40–50. 10.1016/j.worlddev.2015.11.003

[B47] Welsh P. Abba U. Bishop K. Enye C. (2017). *Our Strength Is Not for Hurting’: Engaging Men for Gender equality* [Voices for Change Legacy Paper] Voices for Change. Available at: https://www.sddirect.org.uk/media/1918/1624-v4c-lp-engaging-men-web-1.pdf.

[B48] World Health OrganizationUNICEF (2020). WHO/UNICEF Joint Monitoring Programme (JMP). Available at: https://washdata.org/data/household#!/. (Accessed March 25, 2020).

[B49] Yoon M. Y. (2016). Beyond Quota Seats for Women in the Tanzanian Legislature. Can. J. Afr. Stud./Revue canadienne des études africaines 50 (2), 191–210. 10.1080/00083968.2016.1202849

